# Artificial intelligence in orthopedic regenerative medicine: from design to clinical translational pathways

**DOI:** 10.3389/fcell.2026.1863190

**Published:** 2026-06-09

**Authors:** Tinghui Xu, Wenqian Chen, Yinying Chai, Jiao Hong, Yi Wu, Yichen Xu, Shengliang Qiu, Qiang Luo

**Affiliations:** 1 The First Affiliated Hospital of Zhejiang Chinese Medical University (Zhejiang Provincial Hospital of Chinese Medicine), Hangzhou, Zhejiang, China; 2 School of Medical Technology and Information Engineering, Zhejiang Chinese Medical University, Hangzhou, Zhejiang, China

**Keywords:** artificial intelligence, biofabrication, biomaterial design, deep learning, orthopedic regenerative medicine

## Abstract

Orthopedic regenerative medicine (ORM) addresses musculoskeletal disorders in which effective repair requires coordinated structural reconstruction, biological repair, mechanical adaptation, and functional recovery. These processes generate heterogeneous information across biomaterials, construct design, imaging, intraoperative execution, rehabilitation monitoring, and clinical follow-up. Artificial intelligence (AI) is increasingly relevant for organizing multimodal data and supporting decision-making across regenerative care. This review summarizes current applications of AI in ORM, focusing on regenerative design and fabrication, intraoperative guidance, postoperative monitoring, repair evaluation, and clinical translational pathways. In regenerative design, AI can assist the optimization of material composition, scaffold architecture, biofabrication parameters, and construct performance by linking design variables with biological and biomechanical outcomes. During intervention and recovery, AI-supported systems may improve defect-specific spatial matching, support longitudinal functional assessment, and help identify delayed or unfavorable repair trajectories through integrated analysis of imaging, wearable, and clinical data. The review also discusses translational challenges, including data heterogeneity, limited external validation, algorithmic bias, interpretability, regulatory requirements, and governance constraints. AI may help connect design, intervention, monitoring, and feedback within a continuous analytical workflow, but future progress will require robust datasets, prospective validation, clinically interpretable models, and implementation strategies aligned with regenerative practice.

## 1 Introduction

The restoration of musculoskeletal tissues remains a major challenge in orthopedics, particularly in conditions such as bone defects, cartilage injury, osteochondral lesions, and impaired fracture healing (Bai et al., 2024). These conditions are not defined solely by structural loss, but are frequently accompanied by disruption of the surrounding biological and mechanical microenvironment (Wen et al., 2026; Mathavan et al., 2025). Changes in vascular supply, inflammatory activity, cellular signaling, and local mechanical loading can impair intrinsic repair processes and compromise the durability of treatment outcomes (Chen et al., 2025; Yan et al., 2026; Walk et al., 2025). Conventional orthopedic practice is primarily structured around anatomical diagnosis and procedural feasibility (Pacheco-Brousseau et al., 2026). While this approach is effective in addressing structural defects, it is less capable of explaining why repair outcomes vary substantially across patients and lesion types. Differences in baseline tissue quality, host response, inflammatory status, and mechanical environment all influence how repair progresses over time (Lu et al., 2024; Borrás, 2025; He et al., 2025a). As a result, treatment responses are often difficult to predict using conventional experimental or experience-based approaches alone.

Regeneration can be understood as a process that extends beyond structural repair to include the recovery of biological activity and functional performance. It involves the re-establishment of tissue architecture, restoration of cellular function and vascular support, and the ability of the repaired tissue to sustain physiological loading (Ambrosi et al., 2025; Yang et al., 2024). From a biological perspective, regeneration proceeds through partially overlapping phases of tissue integration, remodeling, and maturation, which together determine the stability and durability of repair. Orthopedic regenerative medicine (ORM) therefore focuses on the coordination of biological and mechanical processes, particularly in complex regions such as osteochondral interfaces, where differences in tissue composition, vascularization, and mechanical properties present additional challenges.

ORM also generates diverse forms of data, including imaging findings, biomaterial characteristics, cellular responses, functional assessments, and longitudinal clinical outcomes (Feng et al., 2024; Sun et al., 2024; Soubrier et al., 2025). Bringing these sources of information together is not straightforward, as relationships between structure, biology, and function evolve over time and do not follow simple linear patterns. Artificial intelligence (AI) is increasingly being introduced into different stages of the regenerative process, including biomaterial design, intraoperative guidance, postoperative monitoring, and outcome evaluation (Razavi et al., 2025; Wang et al., 2023; He et al., 2025b). Its role is not limited to improving efficiency. More importantly, AI enables connections to be made between design decisions, procedural factors, and subsequent recovery patterns, allowing these elements to be analyzed within a more continuous framework (Berni et al., 2023; Kader et al., 2025).

Despite growing interest in this area, current studies often address individual stages of the regenerative process in isolation. A framework that systematically links design, intervention, and recovery, and incorporates feedback from clinical outcomes into earlier stages of decision-making, remains underdeveloped. This review therefore summarizes recent applications of AI in ORM, with emphasis on regenerative design, procedural support, longitudinal assessment, and the key challenges that affect its clinical translation.

## 2 AI in regenerative design and fabrication

### 2.1 Predictive biomaterial design

Biomaterial design in ORM extends beyond the selection of scaffolds with acceptable biocompatibility or mechanical strength. In bone and cartilage repair, construct performance is determined by multiple interacting factors, including material composition, architecture, mechanical properties, degradation behavior, and bioactive cues (Dong et al., 2024; Mao et al., 2024; Lin et al., 2021). These factors influence not only early cell attachment and viability, but also subsequent tissue integration, remodeling, and maturation under local mechanical loading. These interdependencies shape cellular responses and tissue stability, making biomaterial development a complex design problem in which biological and mechanical requirements must be considered together.

AI-assisted modeling offers an alternative to iterative trial-and-error strategies. Conventional experimental approaches typically vary one parameter at a time and evaluate changes in cell behavior or repair performance. This approach remains useful for controlled mechanistic studies, but it is less efficient when scaffold composition, pore architecture, stiffness, degradation rate, and bioactive signals interact with one another. In regenerative settings, modifications in biomaterial design often influence multiple outcomes simultaneously. Changes in matrix composition or stiffness, for instance, may affect cell differentiation, tissue integration, and subsequent maturation (Park et al., 2011; Zhang et al., 2020). AI-based models can help relate desired material performance to underlying design parameters in a more systematic manner, enabling more targeted prioritization of candidate designs.

Different AI models should be selected according to the structure of regenerative design data and the intended design output. For structured experimental datasets, such as material composition, porosity, stiffness, and degradation profiles, tree-based ensemble methods including random forests and gradient boosting can be useful because they relate material or fabrication variables to measurable scaffold properties while retaining some interpretability. In bone tissue engineering, XGBoost and AdaBoost have been used to predict the mechanical properties of calcium hydroxyapatite-reinforced PLA scaffolds (Omigbodun et al., 2024). Random forest regression has also been applied to multidimensional collagen scaffold datasets to identify fabrication variables associated with pore morphology and scaffold connectivity, further illustrating how structured experimental data can inform scaffold design (Nair et al., 2021). In contrast, neural network-based models are more appropriate when the task involves inverse design, spatial architectures, or complex structure–property relationships that cannot be fully described by manually selected variables. Recent work has begun to define model selection according to the structure of the regenerative design task rather than by algorithm type alone. In triply periodic minimal surface scaffold design, machine learning-assisted inverse design has been used to infer structural parameters from predefined mechanical targets, thereby reducing the need for repeated empirical adjustment (Liu et al., 2023). For spinodoid bone scaffolds, a back-propagation neural network combined with a genetic algorithm enabled the search for architectures whose elastic constants and anisotropy more closely matched target bone tissues, with the neural network estimating structure–property relationships and the genetic algorithm searching across candidate architectures (Wang et al., 2025a).

### 2.2 Construct engineering for complex repair

In ORM, biomaterials provide a delivery platform for cells or growth factors and shape the local microenvironment that governs cell behavior, matrix deposition, and tissue integration (Avery et al., 2023; Sithole et al., 2023). This dual role becomes particularly critical in interface-rich lesions such as osteochondral defects, where cartilage and subchondral bone differ markedly in composition, mechanical properties, and reparative capacity. These intrinsic differences make coordinated regeneration difficult to achieve within a single construct. A successful osteochondral construct must therefore support chondral and osseous repair at the same time, while maintaining a stable transition zone that can tolerate mechanical loading. Recent studies have reported scaffold-free osteochondral organoids derived from murine induced pluripotent stem cells that recapitulate both cartilaginous and calcified regions (Limrak et al., 2020). In parallel, modified nanohydrogel scaffolds have been shown to support cartilage and bone regeneration and to self-assemble into osteochondral-like structures without delamination (Wang et al., 2025b). Repair of such complex tissues depends on the coordinated integration of cellular, matrix, and structural components, in which material selection represents only one contributing factor.

The performance of such constructs is closely associated with coupled cellular and matrix responses. To better characterize these processes, AI-based image analysis has been used to convert heterogeneous experimental observations into quantitative descriptions of cell state and microenvironmental response. Image-based learning is particularly relevant for construct evaluation because cellular morphology often reflects early responses to scaffold architecture and matrix cues. High-content imaging studies have shown that machine-learning methods can classify human mesenchymal stem cell (hMSC) states within three-dimensional biomaterial niches by analyzing spatial nuclear organization and other image-derived features (Dh et al., 2016). Morphology-based deep learning work has shown that convolutional models can use cell-shape information to predict hMSC lineage differentiation, including osteogenic differentiation, suggesting that image-derived morphology may serve as a useful readout for early regenerative responses in construct evaluation (Mai et al., 2023).

Building on these approaches, machine-learning-based shape phenotyping has been used to capture morphological response patterns of bone marrow stromal cells across varying microenvironments (Chen et al., 2016). Predictive models have also been developed to estimate biomaterial-induced stem cell lineage commitment and to support early functional evaluation of biomaterials (Zhou et al., 2023). Such models are most informative when they link biomaterial properties with interpretable stem cell responses, particularly lineage commitment and early functional changes (Zhou et al., 2023). Predictive modeling therefore contributes to construct evaluation by connecting material design with biologically meaningful repair responses, rather than merely classifying images or samples.

### 2.3 AI-assisted biofabrication

As regenerative constructs increase in complexity, fabrication has become a key determinant of repair quality. Successful regenerative repair relies on biomaterial selection and biological cue design, as well as their translation into constructs with sufficient spatial precision, structural fidelity, and reproducibility (Ye et al., 2025). This is particularly important for constructs intended to function in load-bearing environments, where small deviations in geometry, pore connectivity, or material distribution may affect later integration and mechanical adaptation. Bioprinting plays a central role in this process by enabling spatially controlled deposition of cells and biomaterials into predefined three-dimensional architectures (Möller et al., 2017). In osteochondral repair, robotic-assisted *in situ* 3D bioprinting has demonstrated high printing accuracy and rapid intraoperative fabrication, with effective cartilage regeneration reported in a rabbit model (Ma et al., 2020). Fabrication therefore represents a critical stage at which regenerative design is either realized in practice or compromised.

Construct quality depends on multiple interacting variables, including extrusion pressure, nozzle geometry, printing path height, and rheological properties, which are difficult to optimize through empirical tuning alone (Klak et al., 2021). Data-driven process control enables a shift from isolated parameter adjustment toward coordinated control of coupled process variables. In extrusion-based printing of Pluronic hydrogels, machine learning has been used to associate nozzle specifications, temperature, path height, and ink composition with printability outcomes (Fu et al., 2021). Recent work has extended this direction to process monitoring and quality control, where data-driven methods support the identification of printing defects and adaptive adjustment of fabrication parameters in real time (Bonatti et al., 2022; Kelly et al., 2025).

**FIGURE 1:**
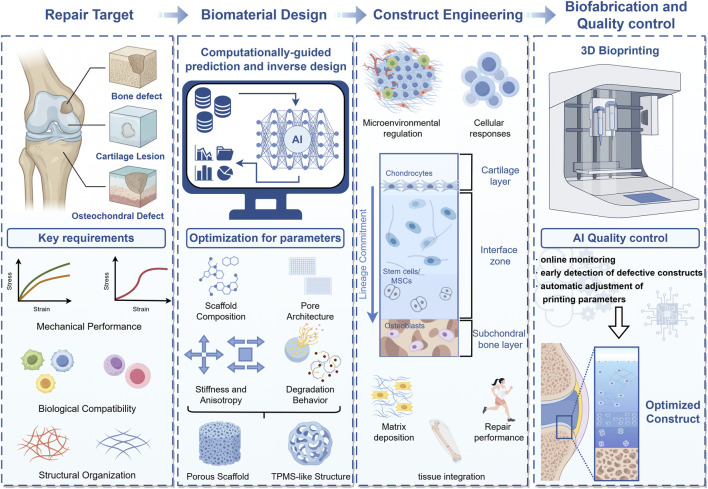
Conceptual framework of data-driven regenerative design and fabrication in orthopedic regenerative medicine. Schematic overview of a data-driven regenerative workflow in orthopedic regenerative medicine, spanning from repair targets to construct fabrication and quality control. Clinically defined defects, including bone, cartilage, and osteochondral lesions, establish key functional requirements in terms of mechanical performance, biological compatibility, and structural organization. These requirements guide the biomaterial design stage, where computationally driven prediction and inverse design strategies are used to optimize scaffold composition, pore architecture, mechanical properties, and degradation behavior. The resulting designs are translated into functional constructs that regulate the local microenvironment and cellular responses, enabling coordinated tissue formation across cartilage and subchondral bone layers. During biofabrication, approaches such as three-dimensional bioprinting are integrated with real-time quality control, allowing online monitoring, early detection of defects, and dynamic adjustment of printing parameters, ultimately supporting the generation of optimized constructs aligned with repair objectives.

The relationship between process variables and construct performance is also being increasingly quantified. Machine learning models can predict key parameters required for high-fidelity printing using outputs such as linewidth and geometric accuracy, thereby linking fabrication settings to printability (Bone et al., 2020). Beyond printability, mechanical behavior at the construct level can also be inferred from imaging features. Compressive responses of printed structures have been predicted using computer vision and neural network-based approaches trained on cross-sectional images (Roach et al., 2021). AI can accelerate screening and monitoring, while physics-based simulation helps interpret whether a printed construct is mechanically suitable for regeneration. More importantly, it helps preserve the intended relationship between construct design, structural fidelity, and subsequent tissue response (Figure 1).

## 3 AI in procedural guidance and repair evaluation

### 3.1 Intraoperative guidance and decision support

In regenerative orthopedic procedures, intraoperative guidance is important because the final execution of a repair strategy occurs within a dynamic surgical field. Even when a construct has been designed before surgery, its reparative effect depends on how accurately it is matched to the actual defect during the procedure. In studies of bone and cartilage defect repair, defect-derived spatial information has been used intraoperatively to guide material deposition and improve geometric matching between the printed construct and the native defect site (Li et al., 2017).

Robotic-assisted and machine-vision-guided *in situ* bioprinting further illustrates this intraoperative role. Rather than serving only as a fabrication technique, *in situ* bioprinting uses surgical-field information to guide where and how cells or biomaterials are deposited within the defect. In cartilage repair, machine vision has been used to support defect segmentation and printing-path guidance, while robotic systems with tool-center-point calibration have improved deposition accuracy during repair procedures (Ma et al., 2020; Lei et al., 2024). These approaches may reduce reliance on intraoperative estimation and support more consistent execution of procedures that require accurate spatial matching between the defect and the repair material.

### 3.2 Postoperative monitoring and recovery evaluation

Postoperative follow-up in orthopedic regeneration can no longer rely solely on clinic visits or isolated measurements, because repair evolves through a gradual interaction between tissue maturation and functional loading. This process involves integration, remodeling, maturation, and functional adaptation, and observations at a single time point often reflect only one stage of this continuum. Early structural filling does not necessarily indicate stable interface integration, and favorable imaging findings may coexist with incomplete biomechanical restoration or limited functional durability (Perdisa et al. 2017; Marmor et al., 2024; Davis et al., 2021). Postoperative evaluation should therefore focus on recovery trajectories rather than on a single favorable measurement.

Digital monitoring provides a way to observe this process more continuously. In cartilage repair, machine learning-augmented near-infrared spectroscopy has been used for *in vivo* follow-up of cartilage defects. By relating spectral information to cartilage thickness and biomechanical properties through convolutional neural networks, this type of analysis supports longitudinal assessment of cartilage tissue status after injury or repair (Sarin et al., 2021). Experimental work using wireless sensors has further shown that longitudinal monitoring of mechanical signals within a regenerative niche can provide information related to subsequent bone repair (Klosterhoff et al., 2020). Regenerative rehabilitation research has also combined implantable strain sensors with subject-specific finite element modeling to estimate local mechanical signals within segmental bone defects and to relate rehabilitation intensity to bone regeneration and functional recovery (Williams et al., 2024). Because longitudinal signals may be influenced by probe contact, sensor stability, loading variability, and measurement noise, AI-based processing and biomechanical modeling are needed to extract signals that remain clinically interpretable during repair monitoring. Repeated measurements can then be organized into healing trajectories, and machine-learning models integrating clinical or imaging-derived variables may help identify robust callus formation, delayed repair, or early risk of non-union (Jin et al., 2025; Yu et al., 2025).

Repair evaluation should further determine whether structural restoration is translated into durable clinical benefit. Pain relief, mobility restoration, joint performance, and patient-reported outcomes often provide a more direct reflection of therapeutic efficacy than imaging alone. In cartilage restoration, machine-learning models using clinical, lesion-related, and intraoperative variables have been developed to predict failure after knee cartilage procedures. Random Forest achieved the best overall performance among several algorithms, while LIME analysis provided patient-specific interpretation of failure risk (Gilat et al., 2024). AI-based models integrating structural information with activity-related data have begun to support functional outcome prediction and provide a more comprehensive estimate of recovery (Ramkumar et al., 2021). Patient-specific digital models may further extend postoperative assessment by combining imaging, motion data, musculoskeletal modeling, and biomechanical simulation to estimate how recovery-related loading affects the repaired site. Orthopedic trauma research has developed a digital twin workflow that integrates patient-specific imaging, motion capture, musculoskeletal modeling, and finite element simulation, supporting the feasibility of individualized biomechanical assessment in orthopedic care (Andres et al., 2025). When postoperative data are linked with intraoperative measurements, such workflows may support a closed-loop system in which recovery patterns inform subsequent surgical planning, construct design, and rehabilitation strategies (Table 1).

**TABLE 1:** AI-supported procedural guidance, monitoring, and feedback-oriented repair evaluation in orthopedic regeneration.

Clinical phase	Ref	Regenerative context	Clinical modality or tool	AI method and regenerative relevance
Intraoperative guidance	Li et al. (2017)	Bone, cartilage, and osteochondral defect repair	3D scanning and *in situ* 3D printing	Digital defect reconstruction and CAD-based geometric matching were used to guide material deposition and improve matching between the printed construct and native defect site
	Ma et al. (2020)	Cartilage regeneration using HAMA hydrogel	Robotic-assisted *in situ* 3D printing	Robot-assisted deposition control and printing-path execution supported intraoperative fabrication and more accurate delivery of regenerative materials to cartilage defects
	Lei et al. (2024)	Cartilage repair by *in situ* bioprinting	Parallel manipulator; machine-vision-guided bioprinting	Defect recognition, printing-path planning, and tool-center-point calibration improved spatial control and consistency of construct deposition within irregular cartilage defects
Postoperative monitoring	Sarin et al. (2021)	Longitudinal monitoring of cartilage defects	Near-infrared spectroscopy during *in vivo* follow-up	CNN-based analysis of spectral data enabled estimation of cartilage thickness and biomechanical properties, supporting longitudinal assessment of cartilage repair or degeneration
	Klosterhoff et al. (2020)	Mechanical monitoring of the regenerative niche during bone repair	Implantable wireless strain sensor	Longitudinal strain-signal monitoring showed that mechanical signals within the regenerative niche can be tracked over time and may inform subsequent bone repair
	Williams et al. (2024)	Segmental bone defect repair and regenerative rehabilitation	Implantable strain sensors with subject-specific finite element modeling	Sensor-derived loading data and finite element simulation were combined to estimate defect-level strain and evaluate how rehabilitation intensity affects bone regeneration and functional recovery
Repair evaluation	Jin et al. (2025)	Prediction of bone repair and fracture healing	Clinical and fracture-related variables	Gradient boosting decision tree and SHAP-based interpretation were used to predict robust callus formation and accelerated fracture healing
	Yu et al. (2025)	Early prediction of impaired bone repair	Micro-CT imaging and bone morphometric parameters	Deep learning segmentation and predictive modeling supported early identification of impaired fracture healing
	Gilat et al. (2024)	Failure prediction after cartilage restoration procedures	Clinical, lesion-related, and intraoperative variables	Random forest, XGBoost, multilayer perceptron, and penalized regression models were compared to predict failure after knee cartilage procedures; random forest showed the best overall performance, while lime provided patient-specific risk interpretation
	Ramkumar et al. (2021)	Outcome prediction after osteochondral allograft transplantation	Preoperative MRI, patient factors, and clinical variables	Machine-learning outcome prediction and SHAP analysis were used to predict clinically meaningful outcomes after cartilage restoration

## 4 AI in clinical translation and implementation

### 4.1 Data heterogeneity and external validation

One limitation of regenerative AI lies in the heterogeneity of the data used for model development and evaluation. Across orthopedic settings, variation may arise from image acquisition, labeling practice, cohort composition, follow-up, and outcome definition (Groot et al., 2021; Buddhiraju et al., 2023). Under such conditions, strong performance in a single-center cohort or an internally split dataset does not guarantee comparable performance in independent populations. External validation studies in orthopedic AI suggest that model performance may change across cohorts and clinical environments, particularly when data distributions differ between institutions or imaging systems (Harper et al., 2025; Olczak et al., 2024). Models intended for clinical use should therefore be evaluated in genuinely independent populations and, when necessary, adapted or recalibrated before broader deployment.

This issue is particularly relevant in ORM, where datasets are often limited in size, multimodal in structure, and vulnerable to imbalance across patient groups. If certain age groups, sexes, ethnic groups, or patients with incomplete follow-up are underrepresented, a model may perform well overall but less reliably in specific subgroups. Future studies should report cohort composition and evaluate model performance across clinically relevant subgroups. Recent recommendations on health AI datasets also emphasize transparency in dataset composition and bias assessment during model development (Alderman et al., 2025).

### 4.2 Model interpretability and clinical actionability

For AI models used in ORM, clinical value depends partly on whether predictions can be interpreted in relation to recognizable biological or biomechanical features. These models are often expected to support treatment decisions during repair rather than provide isolated endpoint predictions. A model may achieve good predictive performance yet remain difficult to use if its output does not clarify which aspect of healing is affected or how management should be adjusted. Explainable frameworks may help address this problem by linking predictions to image regions, motion features, or other clinically intelligible patterns (Tappan et al., 2024; Lysdahlgaard, 2023).

Such interpretability is particularly relevant in regenerative medicine, where repair is evaluated over time and across multiple modalities. When a model indicates an unfavorable course without clarifying whether the concern involves delayed maturation, unresolved inflammation, or poor structural integration, the result becomes harder to verify and less useful for clinical decision-making (Tappan et al., 2024; Breitwieser et al., 2025). Interpretability is also central to trustworthy AI. In high-risk orthopedic procedures, black-box outputs may create uncertainty when AI-supported recommendations influence surgical planning, rehabilitation intensity, or weight-bearing decisions. Models that cannot be related to plausible biological or biomechanical mechanisms may therefore face ethical, legal, and regulatory barriers. Reporting frameworks such as TRIPOD + AI, CONSORT-AI, and DECIDE-AI provide useful guidance for transparent reporting and clinical evaluation of AI-based decision-support systems (Collins et al., 2024; Liu et al., 2020; Vasey et al., 2022).

### 4.3 Data governance and implementation constraints

The development of regenerative AI is constrained by the limited availability and shareability of clinically informative data. Robust models usually require diverse and well-curated datasets, yet data in ORM are often fragmented, institution-specific, and difficult to standardize across sites. Single-site datasets may therefore be insufficient for generalizable model development, while direct cross-institutional pooling is often restricted by privacy and regulatory requirements (Guan et al., 2024). The resulting challenge extends beyond dataset size and includes inconsistency in how clinically relevant information is collected, recorded, and governed, which can impede data integration and model translation.

Privacy-preserving approaches such as federated learning have been explored to support collaborative model development without centralized transfer of raw data. Studies in orthopedic AI suggest that these methods are feasible in selected task-specific settings and may improve model development through multi-site learning (Cina et al., 2025; Liu et al., 2022). However, collaborative training does not eliminate institutional variation in data quality, infrastructure, or dataset scale. In some orthopedic AI tasks, institutions with very small datasets may gain little from federated learning, whereas sites with larger datasets may not consistently outperform local training alone (Kazlouski et al., 2025). These limitations may be amplified when data are non-independent and non-identically distributed across institutions, as may occur with orthopedic imaging protocols, surgical annotations, rehabilitation records, and follow-up schedules. Federated learning studies in medicine show that multi-institutional learning without raw data sharing is feasible, but healthcare and medical imaging studies also indicate that real-world deployment remains constrained by data heterogeneity, uneven local resources, and limited clinical implementation (Sheller et al., 2020; Tzortzis et al., 2025). Common data models, standardized annotation procedures, and shared outcome definitions are therefore needed before federated or multi-institutional learning can reliably support regenerative AI.

Regulatory requirements further shape clinical translation. AI systems that guide diagnosis, treatment planning, intraoperative decisions, or postoperative management may fall within a software-as-a-medical-device framework, depending on their intended use and level of clinical influence. Regulatory analyses of AI-enabled medical devices indicate that approval pathways and device classifications differ across the United States, Europe, and China (Liu et al., 2024; Han et al., 2024). Clinical deployment therefore requires more than predictive accuracy; lifecycle management, performance monitoring, risk control, documentation, and evidence of safety in the intended clinical environment should be considered early in the development of regenerative AI systems (Singh et al., 2025).

## 5 Conclusion

ORM aims to restore damaged musculoskeletal tissues by coordinating structural reconstruction, biological repair, functional recovery, and long-term tissue stability. AI can support this goal by linking regenerative design, procedural execution, and recovery evaluation within a more continuous analytical framework. From biomaterial design and construct fabrication to intraoperative assistance and postoperative monitoring, AI may help connect decisions made before treatment with information generated during intervention and recovery. Future progress should be guided by clinical reliability rather than algorithmic performance alone. Robust multimodal datasets, prospective multicenter validation, interpretable models, and benchmark tasks focused on clinically meaningful regenerative outcomes are needed to support translation. Intraoperative and postoperative data should also be used to refine future construct design, surgical planning, and rehabilitation strategies, rather than serving only as records of treatment response. The broader significance of AI in ORM lies in its potential to support a closed-loop model of regenerative care. In this model, design, intervention, monitoring, and feedback are iteratively connected, allowing regenerative decisions to be adjusted according to biological repair and functional recovery. Realizing this model will require closer alignment among computational methods, clinical workflows, and the biological realities of tissue repair.

## References

[B1] Alderman J. E. Palmer J. Laws E. McCradden M. D. Ordish J. Ghassemi M. (2025). Tackling algorithmic bias and promoting transparency in health datasets: the STANDING together consensus recommendations. Lancet Digital Health 7 (1), e64–e88. 10.1016/S2589-7500(24)00224-3 39701919 PMC11668905

[B2] Ambrosi T. H. Taheri S. Chen K. Sinha R. Wang Y. Hunt E. J. (2025). Human skeletal development and regeneration are shaped by functional diversity of stem cells across skeletal sites. Cell Stem Cell 32 (5), 811–823. 10.1016/j.stem.2025.02.013 40118065 PMC12048286

[B3] Andres A. Roland M. Wickert K. Diebels S. Stöckl J. Herrmann S. (2025). Advantages of digital twin technology in orthopedic trauma surgery – exploring different clinical use cases. Sci. Rep. 15 (1), 19987. 10.1038/s41598-025-04792-w 40481259 PMC12144170

[B4] Avery D. Morandini L. Celt N. Bergey L. Simmons J. Martin R. K. (2023). Immune cell response to orthopedic and craniofacial biomaterials depends on biomaterial composition. Acta Biomater. 161, 285–297. 10.1016/j.actbio.2023.03.007 36905954 PMC10269274

[B5] Bai L. Zhou D. Li G. Liu J. Chen X. Su J. (2024). Engineering bone/cartilage organoids: strategy, progress, and application. Bone Res. 12 (1), 66. 10.1038/s41413-024-00376-y 39567500 PMC11579019

[B6] Berni M. Veronesi F. Fini M. Giavaresi G. Marchiori G. (2023). Relations between structure/composition and mechanics in osteoarthritic regenerated articular tissue: a machine learning approach. Int. J. Mol. Sci. 24 (17), 13374. 10.3390/ijms241713374 37686179 PMC10487849

[B7] Bonatti A. F. Vozzi G. Chua C. K. Maria C. D. (2022). A deep learning quality control loop of the extrusion-based bioprinting process. Int. J. Bioprint 8 (4), 620. 10.18063/ijb.v8i4.620 36404777 PMC9668573

[B8] Bone J. M. Childs C. M. Menon A. Póczos B. Feinberg A. W. LeDuc P. R. (2020). Hierarchical machine learning for high-fidelity 3D printed biopolymers. ACS Biomater. Sci. Eng. 6 (12), 7021–7031. 10.1021/acsbiomaterials.0c00755 33320614

[B9] Borrás C. (2025). Exosomal miR-302b as a novel strategy for reversing cellular senescence and promoting healthy aging. Extracell. Vesicles Circ. Nucl. Acids. 6 (2), 191–194. 10.20517/evcna.2025.24 40852595 PMC12367455

[B10] Breitwieser M. Zirknitzer S. Poslusny K. Freude T. Scholsching J. Bodenschatz K. (2025). AI in fracture detection: a cross-disciplinary analysis of physician acceptance using the UTAUT model. Diagn. (Basel) 15 (16), 2117. 10.3390/diagnostics15162117 PMC12386128 40870969

[B11] Buddhiraju A. Chen T. L. Subih M. A. Seo H. H. Esposito J. G. Kwon Y. M. (2023). Validation and generalizability of machine learning models for the prediction of discharge disposition following revision total knee arthroplasty. J. Arthroplasty 38 (6s), S253–S258. 10.1016/j.arth.2023.02.054 36849013

[B12] Chen D. Sarkar S. Candia J. Florczyk S. J. Bodhak S. Driscoll M. K. (2016). Machine learning based methodology to identify cell shape phenotypes associated with microenvironmental cues. Biomaterials 104, 104–118. 10.1016/j.biomaterials.2016.06.040 27449947 PMC11305428

[B13] Chen M. Liu Y. Cao Y. Zhao C. Liu Q. Li N. (2025). Remodeling the proinflammatory microenvironment in osteoarthritis through Interleukin-1 beta tailored exosome cargo for inflammatory regulation and cartilage regeneration. ACS Nano 19 (4), 4924–4941. 10.1021/acsnano.4c16785 39848926

[B14] Cina A. Tuci M. Pellisé F. Yilgor C. Alanay A. Pizones J. (2025). Simulating federated learning to enable multi-hospital collaboration for lumbopelvic alignment estimation. JOR Spine 8 (4), e70120. 10.1002/jsp2.70120 41112065 PMC12529873

[B15] Collins G. S. Moons K. G. M. Dhiman P. Riley R. D. Beam A. L. Van Calster B. (2024). TRIPOD+AI statement: updated guidance for reporting clinical prediction models that use regression or machine learning methods. BMJ 385, e078378. 10.1136/bmj-2023-078378 38626948 PMC11019967

[B16] Davis S. Roldo M. Blunn G. Tozzi G. Roncada T. (2021). Influence of the mechanical environment on the regeneration of osteochondral defects. Front. Bioeng. Biotechnol. 9, 603408. 10.3389/fbioe.2021.603408 33585430 PMC7873466

[B17] Dhaliwal A. Brenner M. Wolujewicz P. Zhang Z. Mao Y. Batish M. (2016). Profiling stem cell states in three-dimensional biomaterial niches using high content image informatics. Acta Biomater. 45, 98–109. 10.1016/j.actbio.2016.08.052 27590870 PMC5262522

[B18] Dong Y. Li J. Jiang Q. He S. Wang B. Yi Q. (2024). Structure, ingredient, and function-based biomimetic scaffolds for accelerated healing of tendon-bone interface. J. Orthop. Transl. 48, 70–88. 10.1016/j.jot.2024.07.007 PMC11342074 39185339

[B19] Feng Q. Fatima K. Yang A. Li C. Chen S. Yang G. (2024). Multi-modal imaging for dynamic visualization of osteogenesis and implant degradation in 3D bioprinted scaffolds. Bioact. Mater. 37, 119–131. 10.1016/j.bioactmat.2024.03.022 38549773 PMC10972765

[B20] Fu Z. Angeline V. Sun W. (2021). Evaluation of printing parameters on 3D extrusion printing of pluronic hydrogels and machine learning guided parameter recommendation. Int. J. Bioprint 7 (4), 434. 10.18063/ijb.v7i4.434 34805600 PMC8600308

[B21] Gilat R. Gilat B. Wagner K. Patel S. Haunschild E. D. Tauro T. (2024). Evidence-based machine learning algorithm to predict failure following cartilage procedures in the knee. J. Cartil. and Jt. Preserv. 4 (3), 100161. 10.1016/j.jcjp.2023.100161

[B22] Groot O. Q. Bindels B. J. J. Ogink P. T. Kapoor N. D. Twining P. K. Collins A. K. (2021). Availability and reporting quality of external validations of machine-learning prediction models with orthopedic surgical outcomes: a systematic review. Acta Orthop. 92 (4), 385–393. 10.1080/17453674.2021.1910448 33870837 PMC8436968

[B23] Guan H. Yap P.-T. Bozoki A. Liu M. (2024). Federated learning for medical image analysis: a survey. Pattern Recognition 151, 110424. 10.1016/j.patcog.2024.110424 38559674 PMC10976951

[B24] Han Y. Ceross A. Bergmann J. (2024). Regulatory frameworks for AI-Enabled medical device software in China: comparative analysis and review of implications for global manufacturer. Jmir Ai 3, e46871. 10.2196/46871 39073860 PMC11319888

[B25] Harper J. P. Lee G. R. Pan I. Nguyen X. V. Quails N. Prevedello L. M. (2025). External validation of a winning artificial intelligence algorithm from the RSNA 2022 cervical spine fracture detection challenge. AJNR Am. J. Neuroradiol. 46 (9), 1852–1858. 10.3174/ajnr.A8715 39993795 PMC12453477

[B26] He W. Ding F. Zhang L. Liu W. (2025a). *In situ* osteogenic activation of mesenchymal stem cells by the blood clot biomimetic mechanical microenvironment. Nat. Commun. 16 (1), 1162. 10.1038/s41467-025-56513-6 39880808 PMC11779924

[B27] He Y. Chai Y. Liu Y. Chi J. Fei Y. He B. (2025b). RSA-KG: a graph-based rag enhanced AI knowledge graph for recurrent spontaneous abortions diagnosis and clinical decision support. Med Res. 1 (3), 412–423. 10.1002/mdr2.70039

[B28] Jin Z. Chen J. Shan Z. Liu W. Wen Z. Shao H. (2025). Prevalence, risk factors, prediction of robust callus formation and accelerated fracture healing in traumatic brain injury patients: a five-year study. J. Orthop. Transl. 53, 151–160. 10.1016/j.jot.2025.05.011 PMC12240097 40636266

[B29] Kader D. F. Coppola A. Vijay A. Fontalis A. Haddad F. S. (2025). The future of precision orthopaedics: personalized data-driven practice. Bone Jt. Open 6 (7), 836–840. 10.1302/2633-1462.67.Bjo-2025-0056.R1 40669847 PMC12266936

[B30] Kazlouski A. Montoya Perez I. Noor F. Högerman M. Ettala O. Pahikkala T. (2025). Towards practical federated learning and evaluation for medical prediction models. Int. J. Med. Inf. 204, 106046. 10.1016/j.ijmedinf.2025.106046 40706197

[B31] Kelly D. Sergis V. Ventura i Blanco L. Mason K. Daly A. C. (2025). Autonomous control of extrusion bioprinting using convolutional neural networks. Adv. Funct. Mater. 35 (30), 2424553. 10.1002/adfm.202424553

[B32] Klak M. Kowalska P. Dobrzański T. Tymicki G. Cywoniuk P. Gomółka M. (2021). Bionic organs: shear forces reduce pancreatic islet and mammalian cell viability during the process of 3D bioprinting. Micromachines (Basel). 12 (3), 304. 10.3390/mi12030304 33799490 PMC7999205

[B33] Klosterhoff B. S. Kaiser J. Nelson B. D. Karipott S. S. Ruehle M. A. Hollister S. J. (2020). Wireless sensor enables longitudinal monitoring of regenerative niche mechanics during rehabilitation that enhance bone repair. Bone 135, 115311. 10.1016/j.bone.2020.115311 32156664 PMC7585453

[B34] Lei H.-Y. Chen Y.-R. Liu Z.-B. Li Y. N. Xu B. B. Song C. H. (2024). *In situ* bioprinting for cartilage repair using a parallel manipulator. IJB 10 (1), 1437. 10.36922/ijb.1437

[B35] Li L. Yu F. Shi J. Shen S. Teng H. Yang J. (2017). *In situ* repair of bone and cartilage defects using 3D scanning and 3D printing. Sci. Rep. 7 (1), 9416. 10.1038/s41598-017-10060-3 28842703 PMC5572706

[B36] Limraksasin P. Kondo T. Zhang M. Okawa H. Osathanon T. Pavasant P. (2020). *In vitro* fabrication of hybrid bone/cartilage complex using mouse induced pluripotent stem cells. Int. J. Mol. Sci. 21 (2), 581. 10.3390/ijms21020581 31963264 PMC7014254

[B37] Lin Z. Shen D. Zhou W. Zheng Y. Kong T. Liu X. (2021). Regulation of extracellular bioactive cations in bone tissue microenvironment induces favorable osteoimmune conditions to accelerate *in situ* bone regeneration. Bioact. Mater. 6 (8), 2315–2330. 10.1016/j.bioactmat.2021.01.018 33553818 PMC7840811

[B38] Liu X. Cruz Rivera S. Moher D. Calvert M. J. Denniston A. K., and SPIRIT-AI and CONSORT-AI Working Group (2020). Reporting guidelines for clinical trial reports for interventions involving artificial intelligence: the CONSORT-AI extension. Nat. Med. 26 (9), 1364–1374. 10.1038/s41591-020-1034-x 32908283 PMC7598943

[B39] Liu J. Liang X. Yang R. Luo Y. Lu H. Li L. (2022). Federated learning-based vertebral body segmentation. Eng. Appl. Artif. Intell. 116, 105451. 10.1016/j.engappai.2022.105451

[B40] Liu W. Zhang Y. Lyu Y. Bosiakov S. Liu Y. (2023). Inverse design of anisotropic bone scaffold based on machine learning and regenerative genetic algorithm. Front. Bioeng. Biotechnol. 11, 1241151. 10.3389/fbioe.2023.1241151 37744255 PMC10512832

[B41] Liu Y. Yu W. Dillon T. (2024). Regulatory responses and approval status of artificial intelligence medical devices with a focus on China. Npj Digit. Med. 7 (1), 255. 10.1038/s41746-024-01254-x 39294318 PMC11410966

[B42] Lu T. Liu Y. Huang X. Sun S. Xu H. Jin A. (2024). Early-responsive immunoregulation therapy improved microenvironment for bone regeneration *via* engineered extracellular vesicles. Adv. Healthc. Mater. 13 (11), e2303681. 10.1002/adhm.202303681 38054523

[B43] Lysdahlgaard S. (2023). Utilizing heat maps as explainable artificial intelligence for detecting abnormalities on wrist and elbow radiographs. Radiogr. (Lond). 29 (6), 1132–1138. 10.1016/j.radi.2023.09.012 37806069

[B44] Ma K. Zhao T. Yang L. Wang P. Jin J. Teng H. (2020). Application of robotic-assisted *in situ* 3D printing in cartilage regeneration with HAMA hydrogel: an *in vivo* study. J. Adv. Res. 23, 123–132. 10.1016/j.jare.2020.01.010 32099674 PMC7030996

[B45] Mai M. Luo S. Fasciano S. Oluwole T. E. Ortiz J. Pang Y. (2023). Morphology-based deep learning approach for predicting adipogenic and osteogenic differentiation of human mesenchymal stem cells (hMSCs). Front. Cell Dev. Biol. 11, 1329840. 10.3389/fcell.2023.1329840 38099293 PMC10720363

[B46] Mao Z. Bi X. Yu C. Chen L. Shen J. Huang Y. (2024). Mechanically robust and personalized silk fibroin-magnesium composite scaffolds with water-responsive shape-memory for irregular bone regeneration. Nat. Commun. 15 (1), 4160. 10.1038/s41467-024-48417-8 38755128 PMC11099135

[B47] Marmor W. A. Dennis E. R. Buza S. S. Gruber S. Propp B. E. Burge A. J. (2024). Outcomes of particulated juvenile articular cartilage and association with defect fill in patients with full-thickness patellar chondral lesions. Orthop. J. Sports Med. 12 (6), 23259671241249121. 10.1177/23259671241249121 39045351 PMC11265243

[B48] Mathavan N. Singh A. Marques F. C. Günther D. Kuhn G. A. Wehrle E. (2025). Spatial transcriptomics in bone mechanomics: exploring the mechanoregulation of fracture healing in the era of spatial omics. Sci. Adv. 11 (1), eadp8496. 10.1126/sciadv.adp8496 39742473 PMC11694773

[B49] Möller T. Amoroso M. Hägg D. Brantsing C. Rotter N. Apelgren P. (2017). *In vivo* chondrogenesis in 3D bioprinted human cell-laden hydrogel constructs. Plast. Reconstr. Surg. Glob. Open 5 (2), e1227. 10.1097/gox.0000000000001227 28280669 PMC5340484

[B50] Nair M. Bica I. Best S. M. Cameron R. E. (2021). Feature importance in multi-dimensional tissue-engineering datasets: random forest assisted optimization of experimental variables for collagen scaffolds. Appl. Phys. Rev. 8 (4), 041403. 10.1063/5.0059724

[B51] Olczak J. Prijs J. F I. J. Wallin F. Akbarian E. Doornberg J. (2024). External validation of an artificial intelligence multi-label deep learning model capable of ankle fracture classification. BMC Musculoskelet. Disord. 25 (1), 788. 10.1186/s12891-024-07884-2 39367349 PMC11451058

[B52] Omigbodun F. T. Osa-Uwagboe N. Udu A. G. Oladapo B. I. (2024). Leveraging machine learning for optimized mechanical properties and 3D printing of PLA/cHAP for bone implant. Biomimetics (Basel) 9 (10), 587. 10.3390/biomimetics9100587 39451792 PMC11504968

[B53] Pacheco-Brousseau L. Abdelrazeq S. Kelly S. E. Pardo Pardo J. Dervin G. Stacey D. (2026). Total and partial knee arthroplasty *versus* non-surgical interventions of the knee for moderate to severe osteoarthritis. Cochrane Database Syst. Rev. 1 (1), Cd015378. 10.1002/14651858.CD015378.pub2 41494148 PMC12774430

[B54] Park J. S. Chu J. S. Tsou A. D. Diop R. Tang Z. Wang A. (2011). The effect of matrix stiffness on the differentiation of mesenchymal stem cells in response to TGF-β. Biomaterials 32 (16), 3921–3930. 10.1016/j.biomaterials.2011.02.019 21397942 PMC3073995

[B55] Perdisa F. Filardo G. Sessa A. Busacca M. Zaffagnini S. Marcacci M. (2017). One-step treatment for patellar cartilage defects with a cell-free osteochondral scaffold: a prospective clinical and MRI evaluation. Am. J. Sports Med. 45 (7), 1581–1588. 10.1177/0363546517694159 28263667

[B56] Ramkumar P. N. Karnuta J. M. Haeberle H. S. Rodeo S. A. Nwachukwu B. U. Williams R. J. III (2021). Effect of preoperative imaging and patient factors on clinically meaningful outcomes and quality of life after osteochondral allograft transplantation: a machine learning analysis of cartilage defects of the knee. Am. J. Sports Med. 49 (8), 2177–2186. 10.1177/03635465211015179 34048288

[B57] Razavi A. H. Hemmati M. Nafisi N. Mirahmadi A. Laiwala S. Keko M. (2025). Mechanobiology-guided machine learning models for predicting long bone fracture healing across diverse scenarios. Comput. Biol. Med. 196 (Pt A), 110683. 10.1016/j.compbiomed.2025.110683 40628164

[B58] Roach D. J. Rohskopf A. Hamel C. M. Reinholtz W. D. Bernstein R. Qi H. J. (2021). Utilizing computer vision and artificial intelligence algorithms to predict and design the mechanical compression response of direct ink write 3D printed foam replacement structures. Addit. Manuf. 41, 101950. 10.1016/j.addma.2021.101950

[B59] Sarin J. K. te Moller N. C. R. Mohammadi A. Prakash M. Torniainen J. Brommer H. (2021). Machine learning augmented near-infrared spectroscopy: *in vivo* follow-up of cartilage defects. Osteoarthr. Cartil. 29 (3), 423–432. 10.1016/j.joca.2020.12.007 33359249

[B60] Sheller M. J. Edwards B. Reina G. A. Martin J. Pati S. Kotrotsou A. (2020). Federated learning in medicine: facilitating multi-institutional collaborations without sharing patient data. Sci. Rep. 10 (1), 12598. 10.1038/s41598-020-69250-1 32724046 PMC7387485

[B61] Singh V. Cheng S. Kwan A. C. Ebinger J. (2025). United States food and drug administration regulation of clinical software in the era of artificial intelligence and machine learning. Mayo Clin. Proc. Digit. Health 3 (3), 100231. 10.1016/j.mcpdig.2025.100231 40673280 PMC12264609

[B62] Sithole M. N. Kumar P. Du Toit L. C. Erlwanger K. H. Ubanako P. N. Choonara Y. E. (2023). A 3D-Printed biomaterial scaffold reinforced with inorganic fillers for bone tissue engineering: *in vitro* assessment and *in vivo* animal studies. Int. J. Mol. Sci. 24 (8), 7611. 10.3390/ijms24087611 37108772 PMC10144578

[B63] Soubrier A. Kasper H. Miklosic G. Alini M. Jonkers I. Grad S. (2025). A novel intervertebral disc bioreactor system for studying clinically based active dynamic unloading combining biological and biomechanical outcomes. Eur. Cell Mater 50, 1–19. 10.22203/eCM.v050a01

[B64] Sun L. Niu H. Wu Y. Dong S. Li X. Kim B. Y. (2024). Bio-integrated scaffold facilitates large bone regeneration dominated by endochondral ossification. Bioact. Mater. 35, 208–227. 10.1016/j.bioactmat.2024.01.019 38327823 PMC10847751

[B65] Tappan I. Lindbeck E. M. Nichols J. A. Harley J. B. (2024). Explainable AI elucidates musculoskeletal biomechanics: a case study using wrist surgeries. Ann. Biomed. Eng. 52 (3), 498–509. 10.1007/s10439-023-03394-9 37943340 PMC11293275

[B66] Tzortzis I. N. Gutierrez-Torre A. Sykiotis S. Agulló F. Bakalos N. Doulamis A. (2025). Towards generalizable federated learning in medical imaging: a real-world case study on mammography data. Comput. Struct. Biotechnol. J. 28, 106–117. 10.1016/j.csbj.2025.03.031 40212753 PMC11984999

[B67] Vasey B. Nagendran M. Campbell B. Clifton D. A. Collins G. S. Denaxas S. (2022). Reporting guideline for the early-stage clinical evaluation of decision support systems driven by artificial intelligence: DECIDE-AI. Nat. Med. 28 (5), 924–933. 10.1038/s41591-022-01772-9 35585198

[B68] Walk R. E. Broz K. S. Jing L. R.S. Potter C.E. Gonzalez A.T. Beeve (2025). The neurovascular and inflammatory signatures of the degenerating intervertebral disc. Eur. Cell Mater. 54, 65–78. 10.22203/eCM.v054a05

[B69] Wang R. Wang Y. Niu Y. He D. Jin S. Li Z. (2023). Deep learning-predicted dihydroartemisinin rescues osteoporosis by maintaining mesenchymal stem cell stemness through activating histone 3 lys 9 acetylation. ACS Cent. Sci. 9 (10), 1927–1943. 10.1021/acscentsci.3c00794 37901168 PMC10604014

[B70] Wang H. Lyu Y. Jiang J. Zhu H. (2025a). Data-driven inverse design of novel spinodoid bone scaffolds with highly matched mechanical properties in three orthogonal directions. Mater. and Des. 251, 113697. 10.1016/j.matdes.2025.113697

[B71] Wang X. Wu S. Li R. Yang H. Sun Y. Cao Z. (2025b). ROS-activated nanohydrogel scaffolds with multi-factors controlled release for targeted dual-lineage repair of osteochondral defects. Adv. Sci. (Weinh) 12 (20), e2412410. 10.1002/advs.202412410 40156774 PMC12120736

[B72] Wen Y. Zheng X. Li J. He M. Fan D. Zhu X. (2026). Hyperviscous diabetic bone marrow niche impairs BMSCs osteogenesis *via* TRPV2-Mediated cytoskeletal-nuclear mechanotransduction. Adv. Sci. (Weinh) 13 (13), e09056. 10.1002/advs.202509056 41431162 PMC12955904

[B73] Williams K. E. Harrer J. A. LaBelle S. A. Leguineche K. Kaiser J. Karipott S. (2024). Early resistance rehabilitation improves functional regeneration following segmental bone defect injury. Npj Regen. Med. 9 (1), 38. 10.1038/s41536-024-00377-9 39668145 PMC11638264

[B74] Yan X. Wang H. Lu X. Li L. Bai S. Li Z. (2026). Mechanotransduction-driven macrophage polarization *via* Integrin-SRC-STAT6 pathway in distraction osteogenesis. J. Orthop. Transl. 56, 101024. 10.1016/j.jot.2025.10.016 PMC12988528 41836547

[B75] Yang Y. Zhao Z. Qi X. Hu Y. Li B. Zhang L. (2024). Computational modeling of bone fracture healing under different initial conditions and mechanical load. IEEE Trans. Biomed. Eng. 71 (7), 2105–2118. 10.1109/tbme.2024.3361893 38315600

[B76] Ye Y. C. Shao C. Wang Y. Lin F. Su P. Niu Y. (2025). Innovative approach: MRI-Guided fabrication of a biomimetic intervertebral disc scaffold. Eur. Cell Mater. 51, 46–60. 10.22203/eCM.v051a03

[B77] Yu H. Mu Q. Wang Z. Guo Y. Zhao J. Wang G. (2025). A study on early diagnosis for fracture non-union prediction using deep learning and bone morphometric parameters. Front. Med. (Lausanne) 12, 1547588. 10.3389/fmed.2025.1547588 40196347 PMC11973290

[B78] Zhang J. Wehrle E. Adamek P. Paul G. R. Qin X. H. Rubert M. (2020). Optimization of mechanical stiffness and cell density of 3D bioprinted cell-laden scaffolds improves extracellular matrix mineralization and cellular organization for bone tissue engineering. Acta Biomater. 114, 307–322. 10.1016/j.actbio.2020.07.016 32673752

[B79] Zhou Y. Ping X. Guo Y. Heng B. C. Wang Y. Meng Y. (2023). Assessing biomaterial-induced stem cell lineage fate by machine learning-based artificial intelligence. Adv. Mater. 35 (19), e2210637. 10.1002/adma.202210637 36756993

